# A bifunctional DNA binding region in Tn*5* transposase

**DOI:** 10.1111/j.1365-2958.2007.06056.x

**Published:** 2007-12-16

**Authors:** Richard J Gradman, Jerod L Ptacin, Archna Bhasin, William S Reznikoff, Igor Y Goryshin

**Affiliations:** 1 Department of Biochemistry, University of Wisconsin-Madison Madison, WI 53706, USA; 2 Department of Molecular and Cell Biology, University of California Berkeley, CA 94720, USA; 3 Department of Biology, Valdosta State University Valdosta, GA 31698, USA; 4 The Josephine Bay Paul Center, The Marine Biology Laboratory Woods Hole, MA 02543, USA; 5 Epicentre Biotechnologies Madison, WI 53713, USA

## Abstract

Tn*5* transposition is a complicated process that requires the formation of a highly ordered protein–DNA structure, a synaptic complex, to catalyse the movement of a sequence of DNA (transposon) into a target DNA. Much is known about the structure of the synaptic complex and the positioning of protein–DNA contacts, although many protein–DNA contacts remain largely unstudied. In particular, there is little evidence for the positioning of donor DNA and target DNA. In this communication, we describe the isolation and analysis of mutant transposases that have, for the first time, provided genetic and biochemical evidence for the stage-specific positioning of both donor and target DNAs within the synaptic complex. Furthermore, we have provided evidence that some of the amino acids that contact donor DNA also contact target DNA, and therefore suggest that these amino acids help define a bifunctional DNA binding region responsible for these two transposase–DNA binding events.

## Introduction

DNA transposition is a process in which a transposase protein (Tnp) mediates the movement of genetic information (a mobile DNA segment or transposon) within a genome. Transposons are widespread and have been found in every branch of life (Craig *et al*., 2002). These elements play an important role in increasing genetic diversity in a population over time. Furthermore, these elements may carry determinants (such as antibiotic-resistance genes) that allow bacterial populations to quickly adapt to new environmental conditions (Davies, 1994).

Transposase proteins are members of the retroviral superfamily of proteins that include Tnps, retroviral integrases and RAG-1. The catalytic cores of the retroviral superfamily proteins studied in detail are structurally conserved, and the chemical mechanisms by which these proteins function have many similarities (Davies *et al*., 1999; Rice and Baker, 2001). Tn*5* is an excellent model system for studying mobile DNA elements because of the fact that Tn*5* transposition is a relatively simple system requiring only Tn*5* Tnp, Tn*5* DNA (defined by recognition end sequences, ESs), target DNA, Mg^++^ and water, and because extensive genetic, biochemical and structural data exist for this system.

Tn*5* transposition proceeds through a cut-and-paste mechanism involving the breakage and formation of phosphodiester bonds (Goryshin *et al*., 1998). The Tnp and ES containing DNA form a synaptic complex composed of two Tnp protomers and two ESs (Bhasin *et al*., 2000; Davies *et al*., 2000). Then Tnp catalyses the excision of the transposon away from the donor backbone (dbb) DNA by introducing double-strand breaks at each end. The complex containing the excised transposon binds to target DNA in a sequence-biased manner, and this is followed by the insertion of the transposon into target DNA, resulting in a 9 bp duplication (Berg *et al*., 1982). All catalytic reactions take place in the same active site that utilizes three highly conserved amino acid residues (the DDE motif) (Davies *et al*., 2000; Lovell *et al*., 2002). This motif has been discovered in a wide variety of other retroviral integrase superfamily proteins including the RAG1 protein of V(D)J recombination (Kim *et al*., 1999; Swanson, 2001; Brandt and Roth, 2004) and HIV-1 integrase (Rowland and Dyke, 1990; Engelman and Craigie, 1992; Kulkosky *et al*., 1992; Polard and Chandler, 1995).

**Fig. 1:**
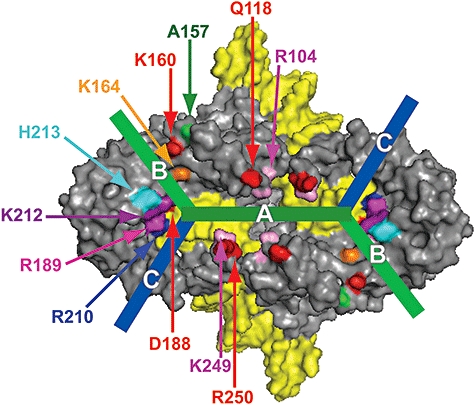
Potential DNA binding regions in Tn*5* synaptic complex. A view of the synaptic complex cocrystal structure. The Tnp surface is represented in grey, while the DNA surface is shown in yellow. The Tnp active site is represented by D188. Labelled positions represent the location of amino acid substitutions in this study with the exception of amino acid A157. Amino acid A157 corresponds to C134 in Tn*10* which was shown to decrease target insertion specificity in Tn*10* transposition (Bender and Kleckner, 1992b). Prospective DNA binding channels are shown as lines and are labelled, A (the prospective 9 bp target site binding region, green), B (light green) and C (blue). Channels B and C represent possible binding regions for flanking target DNA and dbb DNA.

Tn*5* transposition requires a large array of Tnp–DNA binding interactions in order to excise and move the transposon. The X-ray cocrystal structure of Tn*5* Tnp bound to pre-cleaved substrate DNA (Davies *et al*., 2000) provided information on a number of these DNA interactions (Fig. 1). However, as this structure represents one intermediate in the transposition pathway that lacks both dbb and target DNA, it provides no information regarding the disposition of the dbb DNA prior to cleavage and only a partial insight into the location of the target DNA. It is thought that the dbb DNA comes in close contact with the synaptic complex as a result of DNase I footprinting studies showing protection of dbb sequences and because there are dbb sequence biases in synaptic complex formation (Ason and Reznikoff, 2004). The information regarding target binding derives from analyses of the existing crystal structures (Davies *et al*., 2000; Rice and Baker, 2001). The structure contains a positively charged trench between the two active sites that could accommodate 9 bp of DNA, the known size of the target sequence that is duplicated following the last step in transposition. Thus, analysing dbb DNA and target DNA contacts will provide important information regarding the first and last steps in Tn*5* transposition, and will likely illuminate similar phenomena for other retroviral integrase superfamily proteins.

A number of studies have shown that although Tn*5* insertions are random on a large scale, there exist preferred 9 bp insertion sites (Goryshin *et al*., 1998). Tnp insertion preference is a feature that has become apparent for a number of transposons, such as Mu (Haapa-Paananen *et al*., 2002), Tn*10* (Kleckner, 1979) and Tc1 (van Luenen and Plasterk, 1994). In addition to preferred insertion sites, studies of both Tn*5* and Tn*10* have shown that DNA contacts flanking the preferred insertions sites are likely involved in interactions with the Tnp. For Tn*5*, through the sequencing of 24 493 insertion events, it was shown that there was a sequence bias 5 bp long flanking each side of the 9 bp insertion site (Shevchenko *et al*., 2002). The same bias was observed independently through the sequencing of an additional 1960 insertion events (Kang *et al*., 2004). For Tn*10*, through mutagenesis of a known insertion hot spot, the authors defined a 6 bp region flanking the 9 bp insertion site important for target selection (Bender and Kleckner, 1992a). These results indicate that target insertion is dependent on a region of DNA larger than the 9 bp insertion site, and that this entire extended target region may participate through Tnp–DNA contacts. This proposal was supported by recent work on both Tn*5* and Tn*10* transposition in which it was discovered that nicks on the boundaries of a proposed 9 bp target sequences enhanced target specificity (Pribil *et al*., 2004; Whitfield *et al*., 2006). Presumably the nicks lead to an increase in DNA flexibility, thereby reducing the energy needed to create a DNA bend in the target containing DNA, and thus enhanced the Tnp interactions with adjacent nucleotides.

It is formally possible that Tnp binding to target DNA is either competitive, neutral or cooperative with regards to dbb interaction. In fact, current data indicate that different transposition systems manifest different relationships between target and dbb DNA binding. In Tn*7* transposition, the Tn*7* Tnp must capture target DNA before dbb cleavage can occur (Bainton *et al*., 1991; 1993). Phage Mu Tnp is able to bind to target DNA either before or after dbb cleavage (Naigamwalla and Chaconas, 1997). For IS*911* transposition, binding of target is thought to occur by a synaptic complex that exists on a circular DNA intermediate in which the two ES sequences are covalently linked. This circular DNA is formed after dbb DNA release (Loot *et al*., 2002; 2004). Tn*10* Tnp can only bind to target DNA after the dbb–ES cleavage events have occurred (Sakai and Kleckner, 1997). Currently there is no direct evidence for Tn*5* for the interplay between donor DNA, target DNA and the Tnp. However, mechanistic similarities suggest that Tn*5* Tnp behaves identical to Tn*10* Tnp, and is only able to bind to target DNA after dbb cleavage.

In this work, we describe the generation and analyses of a series of site-specific mutations in Tn*5* Tnp that we have used to probe for Tnp–DNA interactions. We isolated a number of Tnp mutations that led to an alteration in target insertion specificity and strand transfer (target capture) activity. Amino acid substitutions of a number of the residues that are involved in Tnp–target DNA recognition are also shown to lead to a reduction in synaptic complex formation in the presence of dbb DNA adjacent to transposon ends. This datum leads to a model in which the Tnp residues altered by these mutations are involved in both target DNA and dbb DNA interactions, and are thus part of a bifunctional DNA binding region. The existence of a bifunctional DNA binding region supports the presumed step-wise binding of the dbb and target DNA sequences; that is, target DNA can only be bound after dbb DNA is released.

## Results

### Generation of potential target specificity mutants in Tn*5* Tnp

**Fig. 2:**
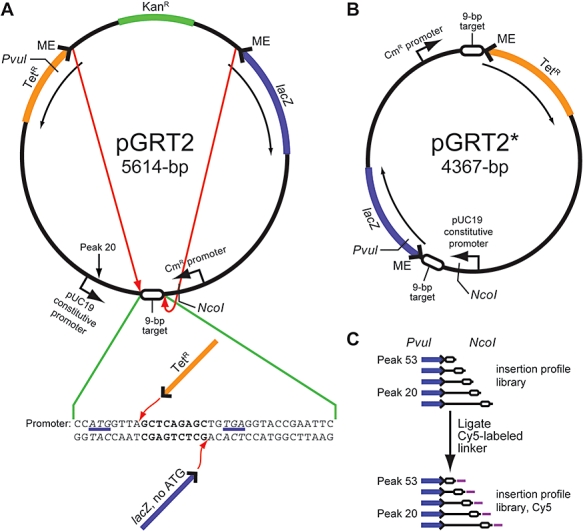
*In vivo*/*in vitro* target specificity assay. A. This assay utilizes the rescue of a Tet^R^ gene to measure transposition frequency and the rescue of a LacZ gene (that is missing a start codon) to measure specific insertion events into a designed, preferable 9 bp target site (GCTCAGAGC). Insertions into this 9 bp target site corresponds to peak 53 in Fig. 3. A second preferable target site is native to plasmid pGRT2 and is noted as peak 20 (see Fig. 3). These experiments were repeated twice at multiple dilutions for each mutant Tnp. B. One possible insertion event out of the library of insertion events is shown. This plasmid represents specific insertion into the 9 bp target site and corresponds to peak 53 in Fig. 3. C. This is a schematic representing the analysis of insertion fragments. After transposition, the library is digested with NcoI and PvuI to generate insertion fragments. The PvuI site is located within the transposon and represents a fixed position. The NcoI site is located within the target DNA. Fragment length is determined by an insertion event relative to the NcoI site and allows us to analyse fragments that contain insertion events that begin near the specific 9 bp target site and extend out to ∼300 bp from the specific target site. After digestion, these fragments were labelled via the ligation of small, 5′-Cy5-labelled DNA to the insertion fragments that allow for visualization of the fragments through PAGE.

A random collection of mutant Tnps was created to screen for target specificity changes. The Tnp library was generated through error-prone polymerase chain reaction (PCR) of a gene that encodes the EK/LP hyperactive variant of Tn*5* Tnp. The library members were screened using one of two reporter plasmids that were essentially identical to pGRT2 shown in Fig. 2. Successful transposition events restore expression of the tetracycline-resistant (Tet^R^) gene, and insertion events into a 9 bp target site, which has been designed to be a preferable target (Goryshin *et al*., 1998), restore the activity of a promoterless *lacZ* (that is also missing a start codon).

**Fig. 3:**
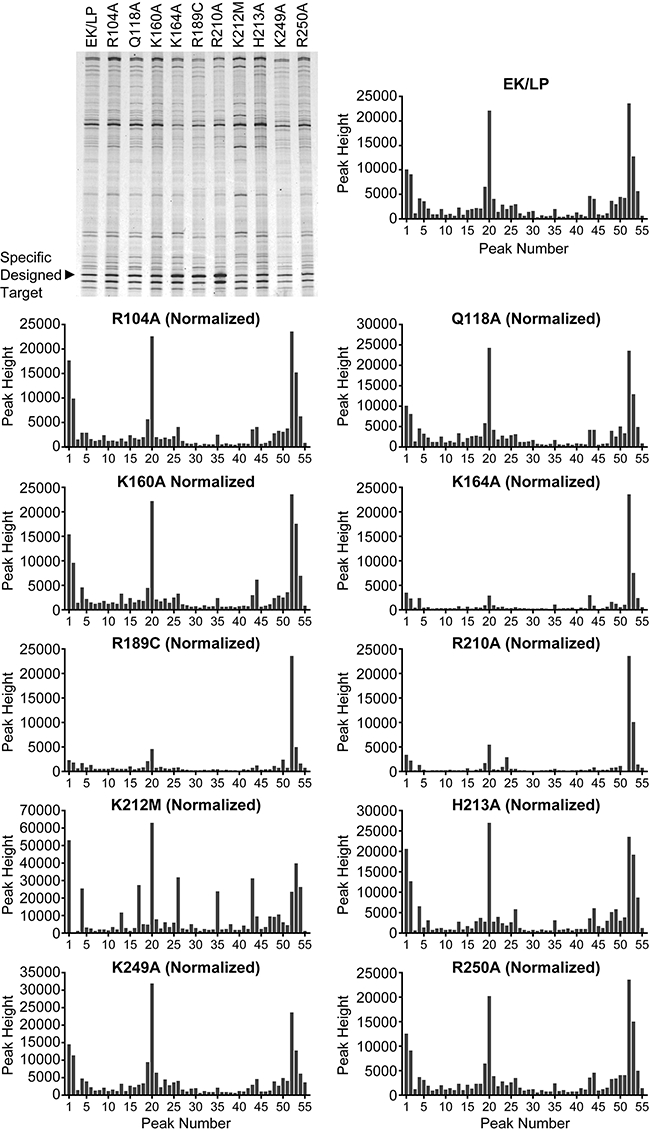
*In vivo*/*in vitro* target insertion profile library. The DNA insertion profile library (as described in Fig. 2C) was analysed via 6% denaturing PAGE. Each insertion profile library generated a pattern of bands representing individual insertion events. The specific insertion into the designed 9 bp insertion site is shown. The insertion profile window is ∼300 bp in length. The peak height of the specific insertion event was normalized for each mutant Tnp to the peak height of the control specific event which corresponds with peak 53. Note the large insertion density present at peak 20. This peak corresponds with an insertion site native to plasmid pGRT2 that was not designed. This native site shares sequence elements with the designed 9 bp target site (GGTTATAGG). The location of this native insertion site is denoted in Fig. 2A. A decrease in the secondary peaks as compared with the specific insertion peak represents an increase in target specificity. K164A, R189C and R210A led to large increases in specificity. K212M led to a slight, but reproducible decrease in specificity and an increase in random insertion events (over the span of this insertion window).

This strategy led to the isolation of two mutant Tnps that altered target insertion specificity: R189C and K212M. The mutation at position R189 led to an increase in target insertion specificity, while the mutation at position K212 led to a reduction in target insertion specificity (data not shown).

It was likely that target DNA contacted more than just the two amino acid positions that were identified in the previously described screen. Therefore, we used our knowledge of the position of these two mutant residues in the X-ray crystal structure of the Tn*5* Tnp synaptic complex (Davies *et al*., 2000), along with sequence alignment data to other IS*50*/Tn*5*-like Tnps (Reznikoff *et al*., 2004), to rationally design an additional set of mutations in Tnp.

The synaptic complex architecture suggests that target DNA binds in a groove that spans the Tn*5* transposase dimeric interface connecting the two active sites. This is in agreement with the distance between the active sites and is the appropriate distance to bind 9 bp of DNA. This groove also possesses several positive charges that makes it ideal for DNA binding (see Fig. 1, channel A) (Davies *et al*., 1999; Rice and Baker, 2001). Using the electrostatic potential of the prospective DNA binding groove as a guide, we looked to sequence alignment data of IS*50*/Tn*5* transposase-related protein sequences (Reznikoff *et al*., 2004) to design mutations. We focused our attention on conserved functional groups as well as conserved charges in the sequence alignment. In the prospective target binding groove, we chose to perform alanine substitution mutagenesis at positions K249 and R250. As a result of X-ray crystal structure analysis, we also decided to introduce alanine substitutions at positions R104 and Q118. These are both positively charged residues located within the prospective target binding groove. These residues are not conserved, but they are located directly across the groove from positions K249 and R250, and seem to structurally mirror these two residues with regards to positioning (see Fig. 1).

The previously described screen isolated two mutant Tnps that affected target insertion specificity outside the target binding groove between the two active sites. Structural evidence suggests that there may be further contacts between Tnp and target DNA, as there are two additional positively charged potential DNA binding grooves that possess conserved residues (Fig. 1, channels B and C). It seemed likely that target DNA would contact residues in one of these grooves and therefore designed our mutations accordingly. We rationally designed specific alanine substitutions based on X-ray crystallographic information at positions K160, K164, R210 and H213.

### Mixed *in vitro/in vivo* assay: identification of amino acid residues that affect target specificity

The above Tnp mutants were purified and the overall target specificity of Tnp mutants was determined using a novel intramolecular *in vivo* assay involving the transposon containing pGRT2 reporter plasmid shown in Fig. 2A. When this reporter plasmid is incubated with Tnp, intramolecular transposition events will occur. These events will produce a library of insertions into the transposon (Fig. 2B). Insertion events that are downstream of the Cm^R^ promoter will rescue Tet^R^, and specific insertion events in one orientation into the designed 9 bp target site will rescue *lacZ* function. Thus this assay allowed for the determination of transposition efficiency as well as the relative preference for the preferred target site for each version of Tnp. Transposition efficiency was determined by comparing total number of Tet^R^ colonies to a no-Tnp control reaction plated on Kan agar (Kan selects for the presence of the plasmid backbone). Target preference is represented by the ratio of blue : total Tet^R^ colonies. These experiments were performed at multiple dilutions in duplicate.

Mutant Tnps K160A, R189C and H213A led to reductions in transposition efficiency greater than fourfold. K164A and R210A led to great reductions in transposition efficiency (77-fold and 31-fold respectively, Table 1). Mutant Tnps R104A, Q118A, K249A and R250A led to slight, but reproducible reductions in transposition efficiency. Unlike the other mutant Tnps studied, K212M was the only mutant Tnp that did not lead to a reduction in transposition efficiency. K212M led to a slight, but reproducible increase in transposition efficiency.

**Table 1:** *In vivo* target specificity results.

	Transposition efficiency	Fold change	Specificity	Fold change
EK/LP	3.1E-4	1.0	0.044	1.0
R104A	1.2E-4	0.39	0.099	2.2
Q118A	2.4E-4	0.77	0.8	1.8
K160A	5.0 E-5	0.16	0.065	1.5
K164A	4.0 E-6	0.013	0.14	3.1
R189C	5.0E-5	0.16	0.16	3.7
R210A	1.0 E-5	0.032	0.36	8.0
K212M	4.2E-4	1.4	0.037	0.8
H213A	7.0 E-5	0.23	0.065	1.5
K249A	2.6E-4	0.84	0.046	1.0
R250A	2.6E-4	0.84	0.046	1.0

Transposition events are represented by the total colony count of the Tet-resistant colonies, and specific events are represented by blue colonies. These were compared with a no-protein control plated on Kan plates to generate transposition efficiency. All of the mutant Tnps resulted in at least a slight, but reproducible net loss of transposition activity except for K212M. The blue : total ratio is used to determine specificity. K164A, R189C and R210A led to significant increases in specificity (increase in the blue : total ratio), while leading to a decrease in transposition efficiency. K212M displayed a slight, but reproducible decrease in specificity (decrease in the blue : total ratio).

Tnp mutants R104A, Q118A, K160A and H213A led to a slight, but reproducible change in target preference (blue : total Tet^R^ colonies). Tnp mutants K249A and R250A led to no detectable change in target preference. Only K212M led to a slight, but reproducible reduction in specificity. Of the mutant Tnps that led to an increase in target preference, K164A, R189C and R210A led to the greatest increases (3.1-, 3.7- and 8.0-fold respectively). These *in vivo* data indicate that K164, R189 and R210 influence target selection and likely contact target DNA. Also, the reduction in specificity in K212M indicates that this position may also contact target DNA.

### Mixed *in vivo/in vitro* assay: denaturing gel electrophoresis analysis of target insertion profile library

The *in vivo* data only analyse one particular insertion event versus a total background of other insertion events, and therefore provide a global analysis of the alteration of target site selectivity. We next wanted to analyse the specific nature of changes in target site selectivity to obtain a better understanding of the specific nature of the changes in target site selectivity (i.e. multiple mutants may lead to an overall increase in target site selectivity with regards to the designed 9 bp insertion site, but may also lead to different levels of insertion into specific secondary sites). In order to visualize specific changes in target site selection, we used an *in vitro* assay. Once again, we utilized the pGRT2 reporter plasmid in Fig. 2 in the same manner as in the *in vivo* assay. After transformation, a sufficient volume of transformed bacteria were plated on agar containing Tet to achieve a density of ∼1000 colonies per plate. All of the colonies were harvested and pooled from each plate and plasmid DNA containing the insertion libraries was purified. The plasmid libraries were digested with PvuI and NcoI to generate insertion profiles of the libraries (Fig. 2C). The lengths of the DNA fragments are variable depending on where each insertion event occurred, and were designed to provide an analysis of insertions over a ∼300 bp window. The insertion profile library was labelled with Cy5 by ligating a short linker to the NcoI site. This labelled library was then analysed via denaturing PAGE and visualized via the Cy5 label.

The results of the *in vitro* target specificity assay are shown in Fig. 3. The specific insertion event at the 9 bp designed target site is indicated and is represented by peak 53. In order to assess changes in target selection, each profile needs to be compared with the profile of the control, EK/LP. In order to analyse these data, each band in each profile was quantified using ImageQuant software. To compare the insertion profile of each mutant Tnp, the peak height for each event was normalized versus the peak height for the specific insertion peak in the designed 9 bp target (peak 53). These normalized peak heights were then plotted and compared with EK/LP (Fig. 3). There is a second preferred target site which corresponds to peak 20 (GGTTATAGG). This site is native to pGRT2 and shares sequence elements with the designed 9 bp insertion site.

From these plots, it is apparent that the insertion profiles for K164A, R189C, R210A and K212M are very different from the profile for the control Tnp, EK/LP. Mutations that led to an increase in target specificity were K164A, R189C and R210A. R210A led to the greatest increase in specificity. These results correspond with the *in vivo* data. Mutant Tnp K212M generated an insertion profile that was more random than the control as shown by an increase in signal for a wide variety of bands over those of the control. K212M was shown to lead to a net decrease in specificity through the *in vivo* analysis, which is supported by these data.

Mutant Tnps, R104A, Q118A, K160A, H213A, K249A and R250A, generated insertion profiles that were similar to that of EK/LP. We believe that the slight differences seen for these mutant Tnps are within experimental error and are not significant.

### Target specificity mutants affect stand transfer (target capture)

The target specificity assay addresses the overall transposition reaction and involves all of the mechanistic steps of transposition. In this section, we address the effects of Tnp mutants on target capture and strand transfer specifically. The strand transfer assay was performed by forming paired-end complexes (PECs) with fluorescently labelled DNA and incubating these PECs with supercoiled pUC19 target DNA. Strand transfer occurs in two mechanistic steps: the first insertion event generates open-circle (with a short tail), or single-end strand transfer products, and the second insertion event generates linear double-end strand transfer products.

**Fig. 4:**
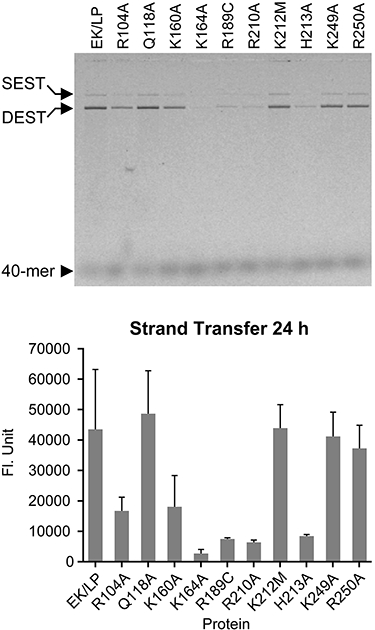
Strand transfer (target capture) assay. The strand transfer reaction generates two products: single-end strand transfer (SEST) product (open-circle DNA) and double-end strand transfer (DEST) product (linear DNA). The amount of DEST product (in fluorescence units, FUs) was determined after 24 h. In comparison with the control, six mutant Tnps are hypoactive for strand transfer: R104A, K160A, K164A, R189C, R210A and H213A.

Compared with the control, the mutants R104A, K160A, K164A, R189C, R210A and H213A displayed hypo-activity for strand transfer with fold decreases in strand transfer of 2.6-, 2.4-, 16-, 5.9-, 6.7- and 5.3-fold respectively (Fig. 4). This is most likely due to defective contacts that these residues have with target DNA. The strand transfer assay actually measures two steps in the Tn*5* transposition reaction: target capture and strand transfer (there is no functional assay for target capture alone in Tn*5* transposition).

The reduction in the strand transfer assay results for R104A and K160A is likely due to a general deficiency in target DNA binding (and not catalysis) as they are far from the active site. These mutant Tnps were not shown to alter target insertion specificity *in vitro*, but did show a reduction in overall transposition efficiency and a slight bias towards more specificity through the *in vivo* assay.

### Target specificity mutants affect dbb DNA recognition

In addition to Tnp–target DNA interactions, we hypothesized that some of the Tnp residues involved in target recognition might also interact with dbb DNA during synaptic complex formation. We addressed this hypothesis through PEC formation assays utilizing native PAGE. The percentage of PECs formed was determined by dividing the quantified shifted DNA by the total amount of quantified DNA present per lane.

**Fig. 5:**
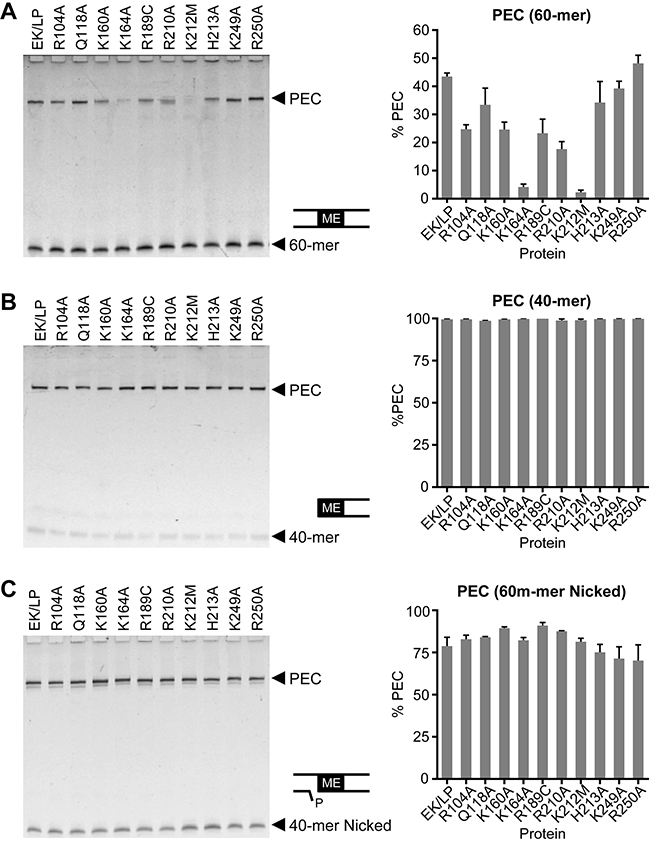
PEC assay. A. PEC formation on full-length (60 bp) substrate is reduced for K164A, R189C, R210A and K212M (note the distributive nature of PECs for R210A). B. PEC formation on a truncated substrate lacking the 20 bp dbb. Mutants that were deficient in forming stable PECs with the 60-mer have had their activity rescued. This suggests that these residues interact with the dbb. C. PEC formation on a substrate containing a nick that mimics the first nick on the transferred strand during dbb–ES cleavage. Total PEC formation is increased and binding activity for the mutant Tnps is restored to control levels. We believe that this is due to the nick providing an increase in the flexibility of dbb DNA.

The initial PEC formation assay utilized labelled 60-mers that possessed the ES flanked by transposon and dbb DNA. In this experiment, the control Tnp was shown to shift ∼44% of the substrate into PECs (Fig. 5A, lane 1). Four of the mutant Tnps that were shown to affect target insertion, K164A, R189C, R210A and K212M, led to a decrease in PEC formation. These mutant Tnps shifted 4%, 23%, 18% and 2% of substrate DNA respectively (Fig. 5A, lanes 5–8). Two mutant Tnps that did not have an effect on target insertion preference also displayed a reduction in PEC formation, R104A and K160A. These mutants each shifted 25% of substrate DNA into PECs. It is also important to note that PECs formed by R210A and K212M were distributive in nature; they possessed a few other minor bands indicating that multiple PEC-like conformations were formed. In these cases, the total amount of substrate DNA shifted was quantified. The distributive PECs for these mutant Tnps most likely result from an impaired ability of the mutant Tnps to bind to the substrate in an optimal conformation.

Synaptic complex formation by the mutant Tnps was further analysed by performing the PEC formation assay with labelled 40-mers that lacked dbb DNA. The results of this experiment are shown in Fig. 5B. This experiment shows that the PEC formation activities of the mutant Tnps are rescued by removing dbb DNA. This experiment was performed at 12°C, and PEC formation reached completion in 5 min. Rescue of synaptic complex formation activity suggests that residues K160, K164, R189, R210 and K212 contact dbb DNA. It is important to note that no distributive PECs were seen for R210A or K212M that formed PECs on pre-cleaved substrate. This indicates that the distributive nature of the PECs is likely due to dbb DNA contacts, or the lack thereof.

In other cases in which impaired PEC formation have been detected (Twining *et al*., 2001), we have found that introducing a nick into the transferred strand that mimicked the first nick in the cleavage reaction would allow enhanced formation of PECs, presumably by compensating for the mutant Tnp defect with an increase in DNA flexibility resulting from the nick. Based upon these observations, we performed the PEC formation assay for our mutants using labelled 60-mer that had the appropriate nick.

The results of this assay are shown in Fig. 5C. These data show similar results to those seen in the 40-mer assay. The nick on the transferred strand rescues the activity of the mutant Tnps and restores EK/LP-like activity levels for the entire series of mutant Tnps. In addition, the distributive nature of the PECs was no longer observed.

## Discussion

Currently, there is only one molecular structure of a full-length transposase bound to ES DNA in a synaptic complex; the cocrystal structure of Tn*5* Tnp bound to pre-cleaved transposon ESs (Davies *et al*., 2000; Lovell *et al*., 2002). Unfortunately, this structure is missing important protein–DNA contacts that play major roles at various steps in the transposition process: contacts between Tnp and dbb DNA (in synaptic complex formation) and between Tnp and target DNA (in target capture). Tn*5* Tnp is a member of the retroviral integrase superfamily of proteins, and shares a similarity in its active site structure architecture and catalytic mechanisms with other proteins in this superfamily, such as other transposases (Mu) and retroviral integrases (ASV and HIV integrases; Davies *et al*., 1999; Rice and Baker, 2001). Thus, being able to determine the rules that govern the DNA contacts in synapsis and target capture would be of significant general scientific value. Therefore, we have defined residues that participate in target and dbb DNA binding through the generation and analysis of site-specific Tnp mutations. We have shown that both DNA binding events involve some of the same Tnp residues, and thus these residues are likely a part of a bifunctional DNA binding region.

### Target DNA interactions with Tn*5* Tnp

It has previously been proposed that target DNA interacts with Tn*5* Tnp through contacts along a basic channel that spans the homo-dimer interface between the active sites (channel A, Fig. 1; Davies *et al*., 2000; Rice and Baker, 2001). However, the existence of preferred base compositions flanking the 9 bp Tn*5* insertion sites (Shevchenko *et al*., 2002; Kang *et al*., 2004) indicates that the flanking regions of target DNA are likely involved in contacts with Tnp beyond the previously described channel.

We have generated a series of mutant Tn*5* Tnps that have been shown to influence target site preference. Substitutions at positions K164, R189 and R210 were shown to lead to a decrease in transposition efficiency while displaying a concomitant increase in target site specificity. Thus, these positions are likely involved in binding target DNA. This in turn suggests that target DNA interacts with channel B shown in Fig. 2, and that the target contact region should include both channels A and B.

In the Tn*10* system, two Tnp mutations were discovered that led to a decrease in target insertion specificity (Bender and Kleckner, 1992b). The authors concluded that the decrease in specificity was due to the mutant Tnps exhibiting a reduction in the preference of certain base pairs as compared with others. Alignment of Tn*5* and Tn*10* Tnp sequences (Haniford, 2002) indicates that the residue changed in one of the Tn*10* target specificity mutations (C134Y) corresponds to A157 in Tn*5* Tnp that is located within the prospective non-specific DNA binding region identified in this communication (channel B, Fig. 1). Additionally, amino acid K137 in Tn*10* is conserved in this alignment with amino acid K160 in Tn*5* that has been shown to influence target (and dbb) DNA binding. This indicates that a non-specific DNA binding region of Tn*10* Tnp may be similarly positioned in the synaptic complex as the Tn*5* Tnp non-specific DNA binding region proposed in this communication. The second Tn*10* target specificity mutation (C249Y) corresponds with an amino acid that is buried in Tn*5* (L283). It is possible that this position is crucial in organizing the fold of this region of the protein.

The importance of the above residues in the final steps of Tn*5* transposition was also studied through the strand transfer assay. The *in vitro* strand transfer assay analyses both target capture and strand transfer. Positions R189 and R210 are near to the active site and led to a reduction in strand transfer activity. Crystallographic evidence suggests that R210 plays a role in clearing the transposon non-transferred strand from the active site after cleavage (A. Czyz *et al.*, unpubl. results). Therefore, a change in this position may indirectly impact target capture by changing the accessibility of the active site. It is likely that R210A Tnp's observed preference for our designed 9 bp target is due to its previously described properties of being a strong target. In this scenario, only strong targets would be able to effectively displace the non-transferred strand DNA that occupies the active site with the defective R210A Tnp mutant. The X-ray crystal structural data (Davies *et al*., 2000) suggest that any role that position R189 would play in clearing the non-transferred strand in strand transfer would be through a backbone interaction that could equally well be performed by alanine. Therefore, we believe that R189 has a direct role in contacting target DNA in target capture.

A mutation at K164 impairs strand transfer, and is located far enough away from the active site so that it likely affects target capture directly and does not affect the catalysis of strand transfer. Mutations at positions R104, K160 and H213 result in a decrease in strand transfer activity. Positions R104 and K160 are far from the active site (position R104 is located in the region of the protein thought to contain the 9 bp target recognition site), which indicates that they affect target capture, and therefore suggests that these residues also likely come into contact with target DNA. Spatially, H213 is located near to the active site, but we believe this amino acid is far enough away from the active site to not contribute to direct catalysis in strand transfer. Therefore, we believe that the reduction in strand transfer suggests that H213 likely comes in contact with target DNA.

We present here a model for target DNA binding. The 9 bp target insertion site binds to the two Tnps in the basic channel that is bound by the active sites and spans the homo-dimer interface (channel A, Fig. 1). The target DNA regions flanking the insertion site wrap around the transposase (channel B, Fig. 1) and come into contact with (or pass near) positions K160, K164, R189 and K212 (or K212M). As mutations of K160, K164, R189 increase specificity, we believe that these contacts form a non-specific DNA binding region that helps contact and position target DNA for transposon insertion. Thus control Tnp contains both residues that make specific DNA contacts and residues that make non-specific DNA contacts, and thus can utilize both specific and random targets although at different frequencies. However, K160A, K164A and R189A mutant Tnps have a reduced ability to bind to random DNA (through the loss of non-specific DNA contacts) which drives insertion into the preferred, specific target site.

### Donor DNA interactions with Tn*5* Tnp

The dbb DNA has been shown to be in contact with Tnp through DNase I footprinting and has been shown to possess sequence bias (Ason and Reznikoff, 2004). These observations led us to conclude that Tn*5* Tnp possesses functional contacts with dbb DNA. This was addressed by using the series of site-specific mutations in Tn*5* Tnp that we used previously to study strand transfer.

We have shown that substitutions at positions K160, K164, R189, R210 and K212 lead to reductions in PEC formation on full-length substrates as well as formation of distributive PECs for R210A and K212M. We believe that the distributive PECs are a result of the improper binding of the substrate DNA, presumably through poor contacts with the dbb DNA, resulting in malformed PECs. Using substrates that lacked dbb DNA, PEC formation activity was restored to control levels for all mutant Tnps, and no distributive PECs were seen. In addition to a restoration of activity, PEC formation activity was shown to be greatly increased for the control Tnp in the absence of dbb DNA as compared with full substrate containing dbb DNA. This suggests that dbb interferes with PEC formation, and that the interference can be partially ameliorated by contacts with residues K160, K164, R189 and K212.

We next attempted to determine the reason for dbb DNA inhibition of synaptic complex formation. A general feature of mobile elements is that proper synaptic complex assembly relies on DNA bending. Previous work has shown that the DNA centred around the cleavage site (the transposon/dbb DNA barrier) is distorted from regular B-form DNA, and is bent 36–48° towards the major groove (York and Reznikoff, 1997; Bhasin *et al*., 2000). We hypothesized that introducing a nick into the dbb DNA would increase the flexibility of substrate DNA (facilitating the bend in the dbb) and lead to an increase in synaptic complex formation. This hypothesis was tested by forming PECs with mutant Tnps on a substrate that possessed a nick that mimicked the first nick on the transferred strand of the cleavage mechanism. This experiment revealed that the mutant Tnps that were unable to form large quantities of PECs using un-nicked DNA (mutations at positions K160, K164, R189, R210 and K212) saw complete rescue of activity as compared with the control Tnp in the presence of nicked DNA. Again, a general increase in PEC formation was also seen in the control Tnp as compared with the un-nicked substrate.

### Evidence for a bifunctional DNA binding region

Our mutational analysis of target insertion preference, the *in vitro* transposition reactions, and our PEC formation assays indicate that a number of positions likely contact both target and dbb DNA. We propose that Tn*5* Tnp possesses a bifunctional DNA binding region adjacent to the active site (Fig. 1). In this model, Tnp contacts dbb DNA, and the target DNA flanking the 9 bp target sequence, in a similar fashion utilizing an overlapping set of amino acid residues. We have shown that target DNA is likely involved in contacts with Tn*5* Tnp at positions R104, K160, K164, R189, K212 and H213 through the alteration of target insertion specificity and/or through alteration of strand transfer activity (likely at the level of target capture) resulting from mutations of these residues. We have also shown that dbb DNA is likely in contact with Tn*5* Tnp at positions K160, K164, R189 and K212 through PEC formation assays. Thus, both of these DNA interactions share a number of contacts. These results led us to conclude that dbb DNA and the region of target DNA flanking the 9 bp insertion site bind to the Tn*5* Tnp at many of the same positions (channel B, Fig. 1).

The bifunctional DNA binding region is supported by other observations as well. Synaptic complex formation and target capture both utilize DNA bending events to mediate their activities (Ason and Reznikoff, 2004; Whitfield *et al*., 2006). We have shown that introducing a nick on the transferred strand at the ES–dbb juncture leads to an increase in PEC formation. The increase in PEC formation is likely due to the increase in DNA flexibility of the ES–dbb juncture, thereby reducing the energy needed to create a DNA bend. In a separate study, nicks introduced into target DNA flanking the 9 bp target insertion site led to an increased target insertion specificity that is also likely to be facilitated by a reduction in the energy barrier for the formation of a DNA bend (Whitfield *et al*., 2006). A similar result was also seen in Tn*10* with regards to an increase of the specificity of strand transfer (Pribil *et al*., 2004). It is important to note that the nick at the end of the 9 bp target sequence will place the nicked DNA in the same spatial position as the first nick on the transferred strand at the transposon–dbb junction, and both nicks lead to an increase in activity (presumably through a facilitation of bending). This indicates that both DNA binding events occur in same region of the protein in a similar fashion.

A model in which dbb DNA and target DNA share a bifunctional DNA binding region would predict that, in Tn*5* transposition, target DNA does not bind Tnp until the transposon has been cleaved free from dbb DNA. This mechanistic detail is seen in other cut-and-paste mechanisms, such as for Tn*10* (Sakai and Kleckner, 1997; Duval-Valentin *et al*., 2004).

## Experimental procedures

### DNA substrates

All DNA oligonucleotides were synthesized and PAGE purified by Integrated DNA Technologies. Where noted (*), DNA oligonucleotides contain a 5′ flurorescein end-label. Sequences of the oligonucleotide substrates follows.

The 60 bp mosaic end (Zhou *et al*., 1998) ME DNA substrate (ME shown in bold) was made by annealing two oligonucleotides, *5′-CTCAGTTCGAGCTCCCAACA**CTGTCTCTTATACACATC**TTGAGTGAGTGAGCATGCATGT-3′ (non-transferred strand) and *5′-ACATGCATGCTCACTCACTCA**AGATGTGTATAAGAGACAG**TGTTGGGAGCTCGAACTGAG-3′ (transferred strand), also labelled on the 5′ end.

The 40 bp ME substrate was made by annealing two oligonucleotides, 5′-PO_4_-**CTGTCTCTTATACACATCT**TGAGTGAGTGAGCATGCATGT-3′ (non-transferred strand) and *5′-ACATGCATGCTCACTCACTCA**AGATGTGTATAAGAGACAG**-3′ (transferred strand), its complement, which is labelled on the 5′ end.

The nicked 60 bp ME substrate was made by annealing three oligonucleotides, the labelled 60 bp ME non-transferred strand oligonucleotide (as previously described), unlabelled 40 bp ME transferred strand (as previously described) and 5′-PO_4_-TGTTGGGAGCTCGAACTGAG-3′. These oligonucleotides hybridize to form a single double-stranded oligonucleotide containing a nick on the transferred strand which mimics the first nick of the cleavage reaction.

The insertion profile library labelling linker (NCLINK) was made by annealing two oligonucleotides, 5′-Cy5-ATAGGACTAATAAACAAA-3′ and 5′-CATGTTTGTTTATTAGTCCTAT-3′. This double-stranded linker contains a single-strand overhang that is complementary to the NcoI restriction site.

### Site-directed mutagenesis and protein purification

DNA manipulations were performed in *Escherichia coli* strain EC100 obtained from Epicentre. DNA PCR primers were obtained from Integrated DNA Technologies. All mutant Tnps constructs in this study contained three hyperactive mutations E54K (Zhou and Reznikoff, 1997), M56A and L372P (Weinreich *et al*., 1994).

Site-directed mutagenesis was performed using the QuikChange site-directed mutagenesis kit (Stratagene) and pGRTYB35 as template plasmid. Resultant constructs were sequenced to verify the presence of the desired mutation and absence of secondary mutations. All Tnp mutants were purified essentially as previously described (Bhasin *et al*., 1999).

### Generation of potential target specificity mutants in Tn*5* Tnp

A library of mutant Tnps was created via error-prone PCR using *Taq* polymerase (Stratagene) of a gene that encodes the EK/LP hyperactive variant of Tn*5* Tnp. The library members were transformed via electroporation into *E. coli* containing one of two reporter plasmids that were essentially identical to pGRT2 (described below). Transposition events produced a library of intramolecular events, and insertion into the 9 bp target sequence rescued *lacZ* function. The resulting colonies were replica-plated onto Luria–Bertani (LB) agar containing Tet and 5-bromo-4-chloro-3-indolyl-β-D-galactopyranoside. Colonies that contained successful transposition events gained Tet resistance and were able to form subcolonies within the footprint of the original colony. Specific transposition events (insertion into the 9 bp target site) were visualized as blue subcolonies. These blue subcolonies were used to identify ‘of interest’ colonies on the original plates. Tnp encoding DNA was harvested from identified colonies using Qiagen mini-preps and was then sequenced.

### *In vitro* transposition target specificity assay

Plasmid pGRT2 was designed to recover and assess the specificity of intramolecular transposition events. Plasmid pGRT2 contains a designed 9 bp preferred target site: 5′-GCTCAGAGC-3′. The Tet^R^ gene has no promoter but is located next to a ME sequence. In order to restore Tet^R^ activity, this gene must be inserted downstream of the Cm^R^ promoter. The LacZ gene lacks a promoter and a start codon. *lacZ* function is only rescued when it inserts specifically into the designed target sequence, thus placing *lacZ* downstream of a pUC19 promoter, and in-framing with a start codon. After incubation of Tnp with the plasmid pGRT2 in reaction buffer at 37°C for 2 h, Tnp was inactivated by treatment with 0.25% SDS at 37°C, and the plasmid products were transformed into *E. coli* EC100 via electroporation. After electroporation, cells were plated onto LB agar containing both tetracycline and kanamyacin, and were allowed to grow for 48 h.

### *In vitro* transposition target specificity assay: target insertion profile analysis

Colonies containing transposition libraries within pGRT2 were harvested and the plasmid DNA was extracted. This DNA was cut with restriction enzymes NcoI and PvuI to isolate a ∼300 bp region near the preferred 9 bp target site. After restriction endonuclease digestion, the DNA was ligated with double-stranded NCLINK linker containing a Cy5 label. The labelled insertion profile library DNA was then treated with Qiagen buffer QG (to remove acidity from the samples), and was then purified by performing Qiagen mini-preps on the samples. The purified samples were then denatured with formamide and heat, and were electrophoresed on a heated 6% denaturing polyacrylamide gel. The samples were visualized using a Typhoon 9410 Variable Mode Imager, and were quantified using Image Quant Total Laboratory Software.

### PEC formation assay

Twenty-five nanomolar fluorescently labelled double-stranded oligonucleotide (60-mer, 40-mer or nicked 60-mer) containing the ME was incubated with 200 nM Tnp in 20 mM HEPES (pH 7.5), 100 mM potassium glutamate and 100 μg ml^−1^ tRNA. For both the full-length 60-mer substrate and the nicked 60-mer, this reaction proceeded for 1 h at 37°C. For the 40-mer substrate lacking dbb DNA, the reaction proceeded for 5 min at 12°C. The reaction was stopped by the addition of cold loading dye (Promega Blue/Orange 6X Loading Dye) and subjected to electrophoresis on a 7% native gel to separate PECs from unbound substrate DNA. PECs were visualized using a Typhoon 9410 Variable Mode Imager.

### Strand transfer assay

Strand transfer capability was determined by incubating 250 nM Tnp with 25 nM labelled 40 bp double-stranded oligonucleotide in 20 mM HEPES (pH 7.5), 100 mM potassium glutamate and 100 μg ml^−1^ tRNA for 1 h at 37°C to form post-cleavage PECs. The reaction mixtures were then incubated at 20°C for 0.5 h. In order to induce strand transfer, magnesium acetate (final concentration of 10 mM) and 15 nM supercoiled pUC19 DNA were added followed by continued incubation at 20°C for 24 h. The reaction was stopped with the addition of cold loading dye (Promega Blue/Orange 6X Loading Dye) and the products of strand transfer (open-circle single-end strand transfer product and linear double-end strand transfer product), and unreacted substrate DNA were separated via electrophoresis on a 2% agarose gel. Strand transfer products were then visualized using a Typhoon 9410 Variable Mode Imager and were quantified using Image Quant Total Laboratory Software.
